# Web-based Intervention to Promote Physical Activity by Sedentary Older Adults: Randomized Controlled Trial

**DOI:** 10.2196/jmir.2158

**Published:** 2013-02-05

**Authors:** A. Blair Irvine, Vicky A Gelatt, John R Seeley, Pamela Macfarlane, Jeff M Gau

**Affiliations:** ^1^ORCASEugene, ORUnited States; ^2^Michael Mac InnovationsEugene, ORUnited States; ^3^Oregon Research InstituteEugene, ORUnited States; ^4^Department of Kinesiology and Physical EducationNorthern Illinois UniversityDeKalb, ILUnited States

**Keywords:** physical activity, older adults, Internet, sedentary, multi-week, exercise

## Abstract

**Background:**

Physical activity (PA) for older adults has well-documented physical and cognitive benefits, but most seniors do not meet recommended guidelines for PA, and interventions are lacking.

**Objectives:**

This study evaluated the efficacy of a 12-week Internet intervention to help sedentary older adults over 55 years of age adopt and maintain an exercise regimen.

**Methods:**

A total of 368 sedentary men and women (M=60.3; SD 4.9) were recruited, screened, and assessed online. They were randomized into treatment and control groups and assessed at pretest, at 12 weeks, and at 6 months. After treatment group participants rated their fitness level, activity goals, and barriers to exercise, the Internet intervention program helped them select exercise activities in the areas of endurance, flexibility, strengthening, and balance enhancement. They returned to the program weekly for automated video and text support and education, with the option to change or increase their exercise plan. The program also included ongoing problem solving to overcome user-identified barriers to exercise.

**Results:**

The multivariate model indicated significant treatment effects at posttest (*P*=.001; large effect size) and at 6 months (*P*=.001; medium effect size). At posttest, intervention participation showed significant improvement on 13 of 14 outcome measures compared to the control participants. At 6 months, treatment participants maintained large gains compared to the control participants on all 14 outcome measures.

**Conclusions:**

These results suggest that an online PA program has the potential to positively impact the physical activity of sedentary older adult participants. More research is needed to replicate the study results, which were based on self-report measures. Research is also needed on intervention effects with older populations.

## Introduction

Physical activity (PA) for older adults increases active life expectancy while limiting the development and progression of disabling conditions and chronic disease [1]. PA is associated with decreased depression [2], improved mental well-being [3,4] and decreased risk of functional decline [5,6]. It enables seniors to maintain their mobility, to improve muscle strength, and to prevent falls [1,7-9]. While results have been mixed, emergent research links vigorous physical activity with reduced risk for cognitive decline [10-12]. The American College of Sports Medicine recommends that PA programs for seniors include endurance, flexibility, strengthening, and balance enhancement exercises, and that seniors engage in 30 minutes of moderate exercise at least 5 days per week [1,8]. Unfortunately, 39% of adults age 65 and older do not meet recommended PA guidelines [8], 33% report no leisure-time physical activity [13], and there is a lack of interventions targeting sedentary behavior [14].

Although the most effective PA intervention mediators are yet to be determined, theory-based behavioral interventions promoting adoption of exercise as a lifetime habit are recommended [6,14-16]. Some studies suggest that participants need to be active for a minimum of 12 weeks to reap the benefits and develop an exercise routine [17-19]. Home-based PA interventions have produced positive results [20-22], while requiring fewer professional resources compared to community or institution-based programs [23].

Computer-technology with multimedia interfaces has the potential to provide cost-effective personalized home PA interventions [6,24,25]. Early research has examined the relative efficacy of various computerized PA approaches including the use of telephone [26-29], print [16,30-34], and handheld computer technology [35]. As new applications and hardware are developed or updated (eg, iPhones, Droids, iPads), more options are becoming available with 4G telecommunications technology. The benefits of interactively tailored interventions has been supported by some [36-38], but not all research [39]; however, interactive programming that tailors the programming to match the users’ personal preferences also is a promising PA approach [40,41]. In sum, new technological developments offer multiple options for individualized PA interventions on desktop computers and mobile devices, but this is still an emerging field of research that has focused so far on young to middle-aged adults [6,16,24,25,42].

Shaping a PA intervention to an older adult audience requires a thoughtful approach because seniors may have decade-old habits and attitudes to change, and they may have functional limitations due to age or medical conditions. Further, based on rates of Internet adoption, seniors may be less prone than younger age groups to adopt new technological approaches, but the tide is changing as baby boomers age and use of the Internet becomes more commonplace. While seniors have been the slowest age group to use the Internet, they have been the fastest growing population segment in recent years, and as of April 2012, 53% of American adults aged 65 and older use the Internet or email, and 70% of them use the Internet daily [43]. Thus, use of computer technology to deliver a senior PA program has increasing potential. In a literature search, however, we could find no research involving stand-alone, theory-driven, Internet PA interventions for seniors that may tap into this potential. Given the success of Web interventions to influence low-fat eating habits [44], tobacco use [45], family caregiver behavior [46], and the exercise habits of sedentary employees [47], among others, we hypothesized that a stand-alone Web-based intervention could influence the PA of older adults as well.

The intervention in this research was based on the theory of planned behavior [48,49], which posits that attitude toward a behavior, social norms, and perceived behavioral control (ie, self-efficacy [50-52]) lead to behavioral intention and change. Considerable empirical evidence supports the significance of self-efficacy in the adoption and maintenance of health-promoting exercise behaviors for adults generally, for example, [53,54] and older adults in particular [55-59]. Thus, the more positive the attitude and subjective norm and the greater the perceived behavioral control, the stronger the intention to perform the behavior [48,49]. Interventions based on this theory have recently been shown to produce large effects on behavior in Web-based interventions [16].

Consequently, the program was designed to provide information and support that would enhance knowledge, attitudes, self-efficacy, and behavioral intentions to participate in exercise activities on a regular basis. Using the criteria recommended for theoretically driven web exercise programs by Doshi and colleagues [14], the intervention included: general assistance, tailored assistance and feedback, self-assessment, and general information, all combined within a gain-framed messaging framework [60,61].

Stage of change theory [62,63] suggests that readiness to change a behavior is predicted by series of stages. These stages include: precontemplation (not considering changing); contemplation (thinking about changing); decision or preparation (definite plans to change); action (beginning change); and maintenance. Encouraging step-wise movement along the continuum of change is thought to lead to enhanced self-efficacy, as well as greater compliance and participation in a behavior change activity. While stage theory is a popular intervention approach in health promotion, it has had mixed results in exercise research. Marcus et al [64] and Calfas et al [65] showed positive intervention effects from stage-matched interventions, but other research has not shown similar effects [21,66,67]. In the research reported here, we viewed stage-matched messages as unnecessary. The program focused on the action-focused messages and social support for all user choices.

In this study, we developed and tested a stand-alone 12-week Internet intervention designed to improve self-reported PA of sedentary older adults. The randomized design (Clinicaltrials.gov NCT01579240) evaluated self-reported changes in exercise across four domains: endurance, stretching, strengthening, and balance. We hypothesized that the intervention would be linked to improvement in the above PA domains and to theoretically relevant mediators of behavior change (eg, attitudes, self efficacy, behavioral intentions) and that user acceptance would be positive. This was a “real-world” effectiveness trial [24,68] in the participants’ setting of choice, as opposed to an efficacy trial in a more controlled gym or laboratory setting.

## Methods

### Intervention Program

The intervention, entitled *Active After 55,* was a multiple-visit Internet program to enhance functional ability, mobility, and physical activity of older adults. Using text and video messages integrated with interactive values clarification and goal-setting activities, it helped users develop a self-tailored exercise plan (eg, by type, intensity, frequency, duration, and schedule) consisting of four activities: endurance, stretching, strengthening, and balance enhancement. Our rationale for a self-tailored approach was based on formative research in this and a previous Web PA study with sedentary individuals [47] and on the theoretical benefits of behavioral control espoused in the theory of planned behavior [50-52]. We also felt that program users being supported and assisted as they set up their own PA prescription might feel ownership, which might improve program engagement and decrease attrition.

The interactive framework was developed in consultation with professionals experienced in the design and implementation of research-based exercise programs for older adults. Care was taken to include only exercises that the participants could do safely on their own with minimal equipment.

*Active After 55* was designed as a browser format, and it did not control the participants’ actions with mandatory linear pathways. The home page had links to *Personal Activity Planning* (ie, developing a personal exercise prescription), *The Health Value of Exercise, Overcoming Obstacles* (ie, dealing with barriers to exercising), *Tracking Progress* (ie, charting activities and frequency of exercise), *Staying Motivated* (ie, tips and personal stories of how individuals made exercise a habit), *Safety Tips* (ie, avoiding injuries), *Disease Specific Recommendations* (ie, tips on exercising with diseases such as arthritis, osteoporosis), and a library with additional related articles and tip sheets. After logging in, participants were encouraged to visit the section on *Personal Activity Planning*, but they were not required or later prompted to do so.

At the initial 1-hour start-up session, *Active After 55* assisted users in designing a personalized PA program. With 11 subsequent weekly sessions, lasting at least 10-15 minutes or more in length, the content logically built on the previous session, with new exercises or advanced challenges of the same exercise added over time. A narrator and personal coach presented video-based educational content with bulleted text teaching points and interactive knowledge-building content. Onscreen titles, bullets, and printable text articles were written at a grade 6-8 reading level. Each exercise was introduced with text instruction and offered optional video demonstrations employing a diverse cast of older adults.

During the first visit, participants were asked to identify their personal goals and their perceived benefits of increased exercise. Users provided information on their recent exercise history and then categorized themselves (ie, Beginner, Intermediate, Advanced) for each of the four activity types. Next, the program helped the user build a tailored weekly exercise plan for each activity type (eg, Beginner stretching: 5 stretches, 3 days per week; Intermediate endurance: 30 minutes per day, 5 days per week). Given the sedentary target population, the program was not designed to provide aerobic exercise. Since we could not control for inaccurate self-categorization of fitness level (eg, sedentary individual selects Advanced for a category), the activities were designed conservatively. That is, Advanced levels were slight increases in frequency or duration from Intermediate levels, and participants were encouraged by text on their printouts to postpone finishing an activity if it seemed too difficult.

For each activity type, users committed to exercises (eg, type of stretches from a list; type of endurance activity from a list) and scheduled the days of the week when they would do each one. For each of the four activity types, users responded to multiple-choice questions about their confidence in achieving their exercise plan for the following week. If they were not confident, they were interactively asked to adjust either the intensity or duration of that exercise (eg, stretching: decrease number of stretches or days per week; endurance: decrease number of days or minutes per day), and they were queried until they interactively expressed confidence that they could meet their weekly commitment. Users also were encouraged to print the schedule for their reference. The printout included personal goals, next week’s exercise plan and blank exercise tracking sheets, guidelines, and safety tips for each activity type. Finally, the session was summarized by the video narrator who extolled the benefits of following the exercise plan and invited the user back in a week for the next session. While participants could visit the website as often as they liked (eg, to read articles or print out personal exercise plans linked to the user’s ID and password), the next program session was available no sooner than 1 week after the completion of the previous session.

At return visits, users were welcomed back and given video and text support for returning. Based on an interactive self-report about success in adhering to exercise commitments from the previous session, users were appropriately praised and encouraged to continue their efforts. For those who reported no progress, the coach’s message was upbeat, offering praise for coming back, and encouragement to try again. At each visit, the user was offered tailored video support on overcoming self-identified exercise barriers (eg, too tired, lack motivation, lack skills, etc). Each week, new educational material was presented to engage users and enhance their knowledge about how to make exercise a habit over time. Based on the user’s self-reported progress and motivation, changes to the exercise plan from the previous week were recommended, if appropriate. As before, the users selected exercise amounts and schedules for each activity and affirmed their confidence to meet the commitments. At the 12^th^ visit, users were encouraged to maintain their exercise program into the future, making it a habit.

### Research Design

**Figure 1:**
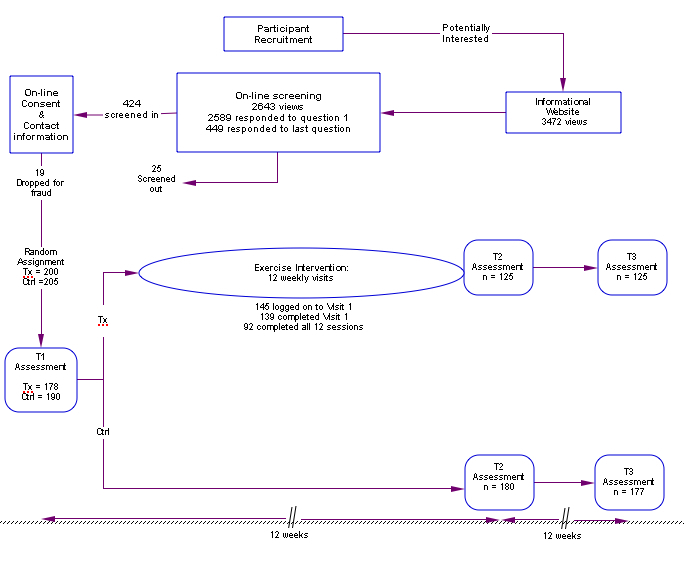
Research design with participation level from recruitment to T3 assessment.

The study was a randomized controlled trial on the Internet with three assessments: pretest (T1), postintervention at 12 weeks after pretest (T2), and 6-month follow-up (T3; see Figure 1). After screening into the study and agreeing to the online informed consent, participants were automatically randomized into a treatment (Tx) group, which used the Internet PA intervention, and a control (Ctrl) group, which did not have access to the intervention.

### Recruitment

After approval by an Institutional Review Board for protection of human subjects (IRB), the study was conducted entirely on the Internet. Participants were recruited via a mixture of online recruitment strategies (eg, listservs, advertising on a website for seniors), flyers, newsletters, and announcements supported by service agencies, senior centers, and worksites. Interested individuals linked to an information website, which offered a link to an online screening questionnaire to determine eligibility (Figure 1). Page views included a total of 3472 on the information website, 2643 on the first screening-questions page, 2589 responses to the first screening item, 449 responses to the last screening item, with 405 individuals ultimately qualifying for the research. This was a rolling recruitment, ie, recruitment was initiated before the intervention was ready for use, with screened-in participants asked to wait, and recruitment continued for 21 days after the T1 assessment and intervention program became available. The average time between participant consent and starting the T1 assessment was 29.1 days (SD 14.8).

### Participant Screening

The online screening questionnaire asked respondents a total of 14 questions about current PA levels (ie, frequency and duration of exercise), desire to exercise more (ie, yes/no), demographics (ie, age, gender, race/ethnicity, employment status, computer use), a working email address, and access to a computer Internet connection. Participants were required to be at least 55 years of age, with a reported desired to engage in more PA. Maximum self-reported exercise levels were: (1) no more than 60 minutes per week of moderate exercise, defined as exercise that increases heart rate, with (2) no bouts of continuous exercise lasting 35 minutes or more. Each respondent answered questions from the 7-item Par-Q [69], which is designed as the minimal standard for entry into a moderate-intensity exercise program.

Individuals who qualified for the study read and agreed to an online informed consent. They then provided contact information, which was checked for fraud before they were randomized by the database into Tx and Ctrl groups. Blinding of the research team to the participants’ research condition was unnecessary.

Our previous Internet research has found a few applicants who attempted to screen-in to a study by providing false information. Consequently, in this study, participant data were checked against our database of about 6000 records from previous Internet study applicants, for fraudulent information (eg, same name or IP address shows inconsistent age, gender, or ethnicity). Screened-in participants providing suspicious data were telephoned, and if the inconsistencies were not resolved, the individual was excluded from the study. A total of 38 were dropped from the study, including 19 before T1, who were not randomized, and 19 after randomization (13 Tx; 6 Ctrl) who were discovered between T1-T3 and were then dropped from the study. Personal privacy was protected with unique user ID and passwords once a participant was accepted into the study and provided contact information. Only passwords provided to Tx participants could link to the intervention.

### Procedures

After completing T1, Tx group participants were mailed a web-enabled CD-ROM (WECD) and emailed log-in information to the *Active After 55* intervention website. Use of the WECD was designed to provide broadband quality video, even from computers with dial-up Internet connections. The WECD, played from the CD-ROM drive of the participant’s computer, contained video with necessary programming code so that specific video elements (eg, an explanation by the coach; an exercise demonstration) were seamlessly integrated into the program presentation, while being controlled by Internet commands from the research host.

Tx group members were asked by a flyer in the WECD mailer to visit the website within a week. One week after a visit to *Active After 55,* an email was sent informing the participant that the next session was now available. The email prompts continued weekly thereafter until the 12 sessions were complete. Twelve weeks after submitting the T1 assessment, all Tx participants who had completed at least Session 1, and all Ctrl participants were emailed a link to the T2 survey. Individuals who had not submitted the T1 assessment or who asked to discontinue participation were dropped from the study. After completion of T2, any Tx participants still in the process of completing the 12 weekly sessions were encouraged to continue using the program until they had completed all sessions.

Twelve weeks after T2, and 6 months after T1, all participants still enrolled in the study were emailed a link to the T3 assessment. After completion of T3, Ctrl participants and individuals who initially were screened out of the study, but who expressed interest in using the site, were emailed a link to access the *Active After 55* website. They were given free access for 6 months.

The protocol for prompting individuals who failed to submit surveys or to complete intervention visits included up to 5 emails over a 1-month time period. They were followed by a single phone call attempting to verify that technical difficulties were not responsible for the lack of participant communication. The individual was then dropped from the study if participation was not re-established. This protocol was developed with the approval of our IRB in other studies. We believe that it allowed for contentious follow-up of consented participants without undo harassments.

All participants were mailed a $25 check after submitting each survey. Participants in the Tx group did not receive a financial incentive to use the intervention website.

### Measures

The T1, T2, and T3 assessments were adapted from our previous Internet research on sedentary factory workers [47], which had satisfactory psychometrics. The items were identical for all participants with the exceptions that demographic questions were asked at T1 only, and Tx group participants were asked to respond to items on website usability and program satisfaction at T2. As noted above, the assessment items were designed to measure program-linked changes on participants’ physical activity, as well as on theoretical constructs that have been shown in previous research to be related to the initiation and maintenance of exercise.

#### Physical Activity Status

Each participant’s self-reported current activity level was measured with 2-item sets addressing the frequency and duration of intentional physical activities that included: (1) cardiovascular activities to increase heart rate (eg, walking briskly, swimming, bicycling, or mowing the lawn), (2) stretching activities to improve flexibility, (3) strength building activities, and (4) balance enhancement activities. For each category, one item asked “In a typical week, how many days do you intentionally…?”, and a pull-down menu offered choices between 0-7 days a week. The second statement asked “How many minutes do you typically … on each of those days?” and a pull-down menu offered choices of from 5-60+ minutes in 5-minute increments. Items were scored to reflect minutes per week of each activity. Scores showed substantial skew, so a log to base 10 transformation was applied.

#### Other Physical Activities

For a sedentary individual, an increase in the number of physical activities, even if they were not categorized as intentional exercise, would indicate an improvement over a sedentary lifestyle. Participants were asked to report on activities they engaged in during the previous week. They were presented with a list of 16 typical physical activities of older adults: yard work, housework, doing exercises, toe raises or stretches, dancing alone as a physical activity, going for a walk for 10 minutes or more, using the stairs instead of an elevator, parking farther away from the store, exercising with others, playing with children, attending activity classes, dancing/square dancing, bowling or other active games, going to a museum, park, or mall, playing golf, and other physical activities. A count of the number of activities engaged in during the previous week was computed for analysis.

#### SF-12

The SF-12 is a 12-item survey that has proven useful in monitoring health outcomes [70]. The SF-12 achieved a multiple R-squared of .918 in predictions of the SF-36 Mental Component Summary Score, which has been validated extensively in research studies. Based on a 4-week recall, the SF-12 items include four physical components: general health (1 item), physical functioning (2 items), role limitations due to physical health problems (2 items), and bodily pain (1 item); and four mental components: vitality (1 item), social functioning (1 item), role limitations due to emotional issues (2 items), and general mental health (2 items). Two summary component scores were created for analysis: Physical Component Summary (Cronbach alpha = .82) and Mental Component Summary (Cronbach alpha = .83).

#### BMI

The Body Mass Index (BMI) has been used as a way to classify sedentary (physically inactive) individuals with an average body composition by the World Health Organization (WHO) [71]. An individual’s BMI is calculated using weight divided by the square of their height. According to the International Classifications (WHO [72]), a BMI greater than 25 is considered overweight and above 30 is considered obese. A limitation of using BMI is that it accounts for weight, but not for differences in body composition [69].

#### Attitudes and Knowledge

The theory of planned behavior suggests that an individual’s attitude and knowledge will shape self-efficacy and intention [48,49]. The line between some attitudinal and knowledge items is blurred, and many attitudinal items might also be considered knowledge items and vice versa. For instance, a statement such as “It’s best to increase activity levels slowly,” might be construed as an opinion or a fact. Therefore, 16 attitudinal items and 5 knowledge items designed to assess program specific content were analyzed as a single-scale score (Cronbach alpha = .91). Items were presented as agree-disagree statements on a 5-point rating scale (1 “Strongly Disagree” to 5 “Strongly Agree”). Attitudinal items addressed opinions and philosophies (eg, the emotional and psychological benefits of exercise; the importance of doing different types of exercise, use of personal strategies to stay active such as sitting less, being active all day, and looking for ways to be active). Knowledge items included information about the benefits of exercise for chronic conditions and avoiding falls, the value of stretching for arthritis, recommended daily activity goals (ie, 30 minutes moderate exercise, most days). For analysis, items were re-coded so that a higher score indicated a more positive attitude or accurate knowledge toward exercise.

#### Behavioral Self-Efficacy

The importance of behavioral self-efficacy to exercise adherence is supported by both social cognitive theory [50,51] and the theory of planned behavior, eg, [73,74]. Five items asked participants how confident were they that in the next month they could, if they wanted to, “be more physically active on a regular basis”, “be physically active most days of the week”, “intentionally do 30 minutes of physical activity in your typical day”, “develop a physical activity plan that would meet your needs”, and “consistently do 4 types of physical exercises (endurance, stretching, strengthening, and balance)”. Responses were measured with a 5-point rating scale (1 “Not at all confident” to 5 “Extremely confident”; Cronbach alpha = .94).

#### Behavioral Intention

The theory of planned behavior suggests that behavioral intentions can predict exercise behavior [75]. Five items asked participants to indicate in the next month how likely they would be to “be physically active most days of the week”, “be physically active for 30 minutes or more a day, 5 days per week”, “develop a physical activity plan that would meet your needs”, “consistently do 4 types of physical exercises (endurance, stretching, strengthening, and balance)”, and “intentionally do 30 minutes of physical activity in your typical day.” Responses were measured with a 5-point rating scale (1 “Very unlikely” to 5 “Very likely;” Cronbach alpha = .93).

#### Motivation

We found no research to adequately measure the motivation of sedentary individuals to exercise, but improvement on this variable should be linked with an increase in PA. Consequently, we adapted the motivation item used by Irvine et al [47] in Internet intervention research on sedentary workers. A single item asked “How motivated are you to be physically active in your daily life?” It was measured on a 5-point rating scale (1 “Not at all motivated” to 5 “Extremely motivated”).

#### Ability to Exercise

A positive change in perceived ability of an individual to perform day-to-day activities was hypothesized to be a measure of improved physical fitness. To assess these capabilities, scales from previous research, eg, [76-79] were adapted into a list of 14 activities that included cardiovascular (eg, walking up two flights of stairs, heavy household chores), strength (eg, lift and carry 10 lb of groceries), balance (eg, balance on one foot for 10 seconds), and stretching (eg, reach into a high cupboard). Participants were asked to rate the difficulty to do each behavior. Reponses were given on a 4-point rating scale (1 “Easy to do,” 2 “Somewhat difficult”, 3 “Difficult,” 4 “Can’t do;” Cronbach alpha = .90)

#### Barriers to Exercise

One goal of the intervention was to change perceptions about possible barriers to participating in physical activity, which research suggests are the reasons many individuals fail to adopt and/or maintain exercise habits, eg, [80,81]. Our previous Internet research on sedentary factory workers [47] compiled a list of 15 barriers to exercise, which showed significant improvement at 30-day follow-up. The list from that study and barriers derived from our unpublished Internet survey of older adults was adapted for this research. The 13 potential barriers included lack of willpower, no one to exercise with, fear of injury, lack of skills, lack of time, bad weather, no safe place, lack of social support, finances, being out of town, too old and out of shape, dislike of sweat, and exercise is boring. Participants were asked to rate how likely each barrier was to prevent them from being physically active in the next week. Reponses were given on a 5-point rating scale (1 “very unlikely” to 5 “very likely”) and combined to form a single-scale score (Cronbach alpha = .88).

#### Stage of Change

If the research intervention was successful, a progression of participants along the continuum of change would be expected. Stage of change (SOC), ie, precontemplation, contemplation, action, maintenance, which assesses an individual’s readiness to adopt new behavior, was measured using four items developed by Marcus, Rossi, Selby, Niaura, and Abrams [82]. A definition of physical activity was provided that included increased heart rate and breathing. Four items were presented: “I am currently physically active (Yes/No)”, “I intend to become more physically active in the next 6 months (Yes/No)”, “I am currently engaged in regular physical activity (Yes/No)”, and “I have been regularly physically active for the past 6 months (Yes/No)”. This instrument had a 2-week Kappa index of reliability of .78 and was correlated with measurements of self-efficacy and intentions [83-85] and with the Seven Day Recall Physical Activity Questionnaire [86].

#### User Satisfaction

User acceptance of the intervention was measured with ratings of perceived satisfaction. Tx group participants responded to additional items relating to their subjective opinions of the *Active After 55* program, including satisfaction, ease of use, helpfulness of the overall information, helpfulness of the articles, and willingness to recommend the program to a friend. For each item, participants were asked to rate their opinions on a 7-point rating scale (1 “Not at all…” to 7 “Extremely...”). A final item asked for opinions on the number of sessions, with a 5-point Likert scale from 1 “Needed many more” to 5 “Far too many”.

## Results

### Participants

A total of 405 participants, including 200 Tx and 205 Ctrl group participants were randomized into the study after consenting, and a total of 368 participants completed the T1 assessment including Tx (n=178) and Ctrl (n=190; Appendix 1) conditions. The sample included 69% female and had a mean age of 60.3 years (SD 4.9). Average BMI was 28.9 (SD 6.7), indicating that the participants, as a group, were considered overweight [69]. They self-identified to be 59% Caucasian, with 41% from other racial and ethnic groups. Most (82%) had at least some college education, 73% had a family income >$40,000/year, and 56.9% were employed. A total of 70% of participants reported using the Internet more than 7 times per week, and 71% emailed more than 7 times per week.

### Baseline Equivalency and Attrition Analysis

The two experimental groups were compared on baseline characteristics and pretest outcome measures. With respect to baseline characteristics, the only significant difference was obtained for race/ethnicity: compared to the Ctrl participants, Tx participants were less likely to be Caucasian, ie, 53% vs. 64%; chi-square (1, N=368) = 4.46, *P*=.035. Given this significant difference, the main outcome analysis included race/ethnicity as a between-subjects factor. The two conditions did not differ significantly on any of the 13 numeric outcome measures or the Stage of Change groups.

Over the course of the study, a total of 84 (62 Tx; 22 Ctrl) of the 405 randomized participants were unresponsive to repeated prompts and were dropped from the study, and 19 participants (13 Tx; 6 Ctrl) were removed as fraudulent during the 6-month period between T1-T3 assessments. Of the Tx group participants, only 145 of the 178 who submitted the T1 assessment logged on to initially use the intervention, and 6 of those participants did not complete Visit 1. A total of 92 (73.6%) of those completing T3 assessments (ie, 51.7%) from the T1 Tx group completed all 12 sessions.

Thus, out of the 178 Tx Group participants at T1, 125 (70.2%) eventually remained in the study to T3. A total of 305 participants (125 Tx group; 180 Ctrl group) submitted a T2 assessment, and 302 (125 Tx group; 177 Ctrl group) submitted a T3 assessment. Overall, T1-T3 attrition was (368-302)/368 = 17.9%.

A significantly higher attrition rate was obtained for the Tx condition compared to the Ctrl condition with 30% vs. 7%; chi-square (1, N=368) = 32.84, *P*<.001. In addition, significantly higher rates of attrition were obtained for male vs. female participants with 25% vs. 15%; chi-square (1, N=360) = 4.51, *P*=.034, race/ethnic minority vs. Caucasian participants at 26% vs. 12%; chi-square (1, N=368) = 32.84, *P*<.001, and those who reported less frequent baseline computer usage, ie, 1-2 times per week = 46%, 3-4 times per week = 44%, 5-6 times per week = 12%, 7 times per week = 14%, 8 or more times per week = 15%; chi-square (4, N=361) = 20.83, *P*<.001. Participants who dropped out of the study after T1 (n=65) were also compared to those who continued participating (n=302) on the pretest outcome measures. Compared to the participants who completed either T2 or T3 assessments, those who dropped out had significantly lower means on the attitudes/knowledge scale at 3.8 vs. 4.1; *t* (365) = 3.26, *P*=.001, ie, attriters had poorer attitudes/knowledge at T1 and significantly higher mean levels for the barriers to exercise scale at 2.7 vs. 2.4; *t* (365) = 2.86, *P*=.005. However, no condition-by-attrition interactions were found to be significant for any of the T1 measures, ie, attriters did not differ across experimental condition on the T1 measures.

### Missing Data and Imputation

Rates of missing study outcomes ranged from 0-1% at T1, 18-21% at T2, and 19-22% at T3. The full-information maximum likelihood estimators assume data are at least missing at random (MAR). It is not possible to know for sure that data are MAR because information about the value of the missing data is not available. However, given the abovementioned significant associations between attrition and study outcomes at baseline, the MAR assumption appears less tenable. Therefore, the main outcome analyses were conducted with (1) available data (ie, “complete cases”, n=294 to 300, dependent outcome), and (2) one fully-imputed dataset that included all 368 study participants. Since the inclusion of additional predictors in the imputation model can reduce bias and make the MAR assumption more plausible [87-89], in addition to the all study outcomes, the imputation model also included all study demographic characteristics.

Sequential regression multiple imputation (SRMI [90]) was used to generate the dataset using the IVEware software V0.2 [91]. SRMI specifies a multivariate model by separate conditional models for each incomplete variable allowing for imputation of variables with different distributional properties. For the current study, three models were specified: a normal linear regression model for continuous variables, a logistic regression model for binary variables, and a generalized logit regression model for variables with more than two categories.

Results indicate that the available data approach and imputed data approach resulted in a similar pattern of results. Following the intent-to-treat approach, results from the imputed model are reported below.

### Program Usage

As mentioned, 125 (70%) of the original Tx group remained in the 12-week study. The mean number of visits to the website for these individuals was 15.2 visits (SD 9.02). The mean total time spent using the program summed across all visits was 123.4 minutes (SD 185.98), and the mean time spent per visit was 9.66 minutes (SD 10.48). Participants each accessed an average of 2.92 (SD 4.30) program segments designed to help overcome specific perceived barriers to exercise.

### Pretest-Posttest Change

A 2 x 2 (condition by race/ethnic minority status) MANCOVA was conducted on the posttest outcome measures in which the pretest outcome measures were included as covariates. The dependent measures included: (1) physical activity measures, (2) SF-12 physical and mental composite measures, (3) BMI, and (4) psychosocial measures (Appendix 2).

An overall multivariate model was tested at posttest, followed by univariate models for each outcome measure. Partial eta-square was used as the estimate of the effect size; values of .01, .06, and .14 represent small, medium, and large effect sizes, respectively [92]. The multivariate model at posttest was significant in which the Tx participants were found to have large gains compared to the Ctrl participants, *F* (14, 337) = 4.81, *P=* .001, eta-square = .17 (Table 1). As can been seen in Table 1, the Tx Group differed significantly from the Ctrl participants on 13 of the 14 outcome measures. The only measure not showing significant T1-T2 change was BMI. The outcome measures with medium effect sizes or larger include cardiovascular exercises min/wk (eta-square = .07), stretching exercises min/wk (eta-square = .07), strength exercises min/wk (eta-square = .11), balance exercises min/wk (eta-square = .09), number of activities (eta-square = .07), behavioral intentions to exercise (eta-square = .10), and motivation to exercise (eta-square = .06). Neither the multivariate main effect for race/ethnicity nor the condition-by-race/ethnicity interaction effect was significant.

**Table 1:** ANCOVA results for the outcome measures.

Outcome measure/ Condition	T1-T2 condition effect			T1-T3 condition effect		
	*F* test	*P*	Eta^2 a^	*F* test	*P*	Eta^2 a^
Cardiovascular activities	26.32	<.001	.067	19.38	<.001	.050
Stretching activities	25.71	<.001	.070	23.36	<.001	.060
Strengthening activities	42.70	<.001	.105	19.03	<.001	.050
Balance activities	37.26	<.001	.092	32.37	<.001	.081
Activities min/wk	25.4	<.001	.068	17.98	<.001	.049
SF-12 physical	5.19	.023	.015	7.56	.006	.021
SF-12 mental	11.41	.001	.032	9.51	.002	.026
BMI (kg/m^2^)	1.04	.309	.003	4.42	.036	.012
Attitudes/Knowledge	10.16	.002	.028	8.57	.004	.024
Self-efficacy	9.08	.003	.025	13.05	.001	.036
Behavioral intentions	38.99	<.001	.1	23.38	<.001	.063
Motivation to exercise	21.22	<.001	.057	22.47	<.001	.06
Ability to exercise	4.14	.043	.012	6.85	.009	.019
Barriers to exercise	8.67	.003	.024	8.26	.004	.023

^a^ Partial eta-square (effect size): .01 small, .06 medium, .14 large.

### Pretest–Follow-Up Change

To examine the maintenance of program effects at follow-up, an overall 2 x 2 MANCOVA model was tested comparing the two conditions on the follow-up outcome measures, controlling for pretest measures, followed by univariate ANCOVA models. The multivariate model at follow-up was significant in which the Tx participants were found to maintain large gains compared to the Ctrl participants, *F* (14, 337) = 3.08, *P*<.001, eta-square = .11. The Tx group differed significantly from the Ctrl participants on all of the 14 outcome measures (see Appendix 2). The outcome measures with medium effect sizes or larger include stretching exercises min/wk (eta-square = .06), balance exercise min/wk (eta-square = .08), behavioral intentions to exercise (eta-square = .06), and motivation to exercise (eta-square = .06). Although there was a significant multivariate main effect of race/ethnicity status with *F* (14, 337) = 1.96, *P*=.020, eta-square = .08, the condition-by-race/ethnicity interaction effect was not significant. Compared to race/ethnic minority participants, Caucasian participants reported significantly lower follow-up mean scores for self-efficacy at 3.6 vs. 3.9; *F* (1, 350) = 5.05, *P*=.025, eta-square = .01, behavioral intentions at 3.6 vs. 4.0; *F* (1, 350) = 5.39, *P*=.021, eta-square = .02, and motivation at 3.3 vs. 3.8; *F* (1, 350) = 12.05, *P*<.001, eta-square = .03.

### Stages of Change Analysis

Stage of change groupings are compared in Table 2, and it is curious that even though all of the participants were screened to be at a maximum of 40% of recommended minimums for weekly exercise [7], 29% of Tx and 21% of Ctrl participants reported being in the maintenance stage at pretest. The two conditions were compared using contingency table analyses at pretest, posttest, and follow-up. They did not differ significantly at pretest, but significant effects were obtained at posttest and follow-up. Compared to Ctrl participants, a larger proportion of Tx participants were found to be in the Action and Maintenance stages at both posttest and follow-up.

**Table 2:** Stages of change groups by condition at T1, T2, and T3.

		Pre-contemplation		Contemplation		Preparation		Action		Maintenance	
		N	%	N	%	N	%	N	%	N	%
**T1^a^**											
	Treatment	3	2	91	52	24	13.6	7	4	51	29
	Control	3	2	97	52	37	19.8	11	6	39	21
**T2^b^**											
	Treatment	–	–	14	11	19	15.4	28	23	62	50
	Control	8	5	57	33	24	13.9	19	11	65	38
**T3^c^**											
	Treatment	1	1	16	13	15	12	13	10	80	64
	Control	4	2	46	26	30	17	17	10	79	45

^a^ Chi square (4, N=363) = 5.12, *P*=.275.

^b^ Chi square(4, N=296) = 28.79, *P*=.001.

^c^ Chi square (4, N=301) = 13.61, *P*=.009.

### Dose-Response Analysis

A dose-response analysis was conducted to examine whether the level of exposure to the program was significantly associated with pretest-posttest change in the outcome measures for the participants assigned to the treatment condition. A composite dose measure was created by standardizing the total time spent across all sessions, the number of page views, and the number of sessions, and computing the mean value of the three standardized scores (Cronbach alpha = .79). A composite outcome measure was created by calculating pretest-posttest gain scores for each of the 13 outcome measures, standardizing each gain score, and computing the mean value of the standardized gain scores (Cronbach alpha = .85). The correlation between the composite dose measure and the composite gain score was significant, *r*=.22, *P*=.014. Thus, a higher level of program utilization was associated with significantly greater change in outcome, which provides further support for the internal validity of the study.

### User Acceptance

On a 7-point scale (eg, not at all satisfied – extremely satisfied), participants reported they were quite satisfied with the program (M=5.6, SD 1.3), the program was very easy to use (M=5.9, SD 1.2), the overall information was very helpful (M=5.9, SD 1.2), the articles provided by the program were very helpful (M=5.7, SD 1.3), and they would be very likely to recommend the program to a friend or family member (M=5.7, SD 1.4). On a 5-point scale to determine opinions about the number of sessions, 89 participants (70%) rated “Just Right,” and 27 (21%) rated “A few too many”.

## Discussion

This randomized effectiveness trial to evaluate the *Active After 55* Web program indicates that the intervention positively impacted the physical activity of sedentary older adults, and it was well received. The hypotheses were that the intervention would be linked to changes in the exercise domains of endurance, stretching, strengthening, and balance and that it would be linked to theoretically relevant mediators of behavior change. The findings were very consistent across an array of measures, with a large multivariate effect size at posttest and a medium multivariate effect size at 6-month follow-up. The Tx group showed significant improvement on 13 of 14 outcomes at posttest and on all 14 outcomes at follow-up (Table 1). Taken together, the results suggest that the intervention had an impact on the self-reported PA of this sample of sedentary older adults, a group that might be expected to be difficult to change.

The strength of the results presented here may actually be diluted somewhat by the measurement process. Treatment subjects who were late completing courses or had stopped taking them were exposed to relatively less of the intervention before the T2 assessment. Additionally, the Ctrl group results generally improved over time, possibly indicating that the assessment process might have brought about reactive effects. Just being exposed to those questions might have sensitized the Ctrl group to improve their level of physical activity and related cognitions.

While defining and identifying a sedentary population of older adults is an inexact science, the screening was designed to disqualify any applicant who engaged in moderate exercise for more than 60 minutes per week, which is 40% of the minimum recommended level [1,7]. The mean BMI value of 28.9 (kg/m^2^) for the participants (Appendix 1) is in the “overweight” category [93]. While being overweight does not of itself indicate that an individual is sedentary, excess weight is often linked with insufficient exercise [7,94,95]. Thus, those who tested the *Active After 55* program seemed to have at least one of the characteristics of sedentary users for which it was designed.

The fact that 92 of the 139 participants (66%) who completed Visit 1 finished all 12 sessions without payment to do so, suggests that the PA intervention was engaging enough for a majority to follow through for 12 weeks. This is a very positive sign, but we found no attrition results from comparable Web research against which to measure our results. As indicated by the dose-response analysis, greater program utilization resulted in greater change in outcomes, which has been reported by other [47,96], but not all [97] Internet PA studies. However, understanding the dose response relationship between improved outcomes and program engagement is complicated by participant attrition [24].

Dropouts in this study had lower attitude/knowledge scores about the benefits of exercise, and they had higher perceptions of barriers to exercise. While the results presented here must be viewed cautiously until validated by other research, they might offer a hint as to why sedentary individuals fail to start to engage in a Web PA program. Other research attributes attrition from PA programs to unrealistic participants’ expectation [98], demographics, and physical characteristics [99], low intention to change [32], time requirements [100], and various perceived barriers to exercise [101]. An alternative supposition for dropouts related to Internet PA programs is offered by the work of Christensen and colleagues, which suggests that some individuals, described with terms such as *e-attainer* [102] or *one hit wonders* [103], benefit from only brief exposure to Internet interventions. That is, participants meet their own Web program participation goals, which do not match those of the researcher(s). Thus, imputing baseline levels for dropouts or looking only at participants who complete a program may confound the dose-response relationship, which also might be related to participant motivation or program engagement [24]. More research is clearly needed to examine the influence of different intervention components on engagement and effectiveness with PA websites [104,105], which might help tease out techniques to improve outcomes and decrease attrition [24].

This research also presents data on recruitment success using an informational website and automated online screening (Figure 1). While 3472 views of the informational website led to 2589 responses to the first screening question, only 449 (17.3%) respondents completed the questionnaire. Why more potential participants did not complete the 14-item questionnaire, which did not request sensitive information and was designed to require less than 5 minutes to complete, is unclear.

Also of potential interest to other researchers is the incidence of fraud reported here. Of the 424 individuals who screened in and agreed to the informed consent, 19 were dropped because of fraudulent information before being randomized, and another 19 subjects were discovered to be fraudulent and were dropped between T1-T3. Thus, 9% of those who screened in provided false information. We have experienced roughly similar numbers in other Internet studies, causing us to set up our fraud database to cross-check participant information. Some individuals are repeat offenders, and we even had one fraudulent participant in another study complain to us when confronted on the telephone that removal from a study constituted mistreatment by us because she said, “I’m only trying to make a living.” We believe that the potential for fraudulent participation in research studies on the Internet is an important issue, but we are aware of no research into the frequency of occurrence or steps to minimize it.

### Limitations

The current results must be viewed cautiously because we have no evidence that the participants actually engaged in PA or provided accurate information. Additionally, some of the measures were not validated in other research, physical functionality was not measured beyond the SF-12 physical sub-scale, and the follow-up period was somewhat limited (ie, 6 months). Follow-up studies of 1-2 years, using participant exercise logs and verifiable measures of PA (eg, treadmill testing; 6-minute walk testing), and functionality (eg, Physical Functional Performance Test [106]; AM-PAC Physical Mobility, and Personal Care scales [107]) would provide greater confidence in the intervention effects. Also, the assessment measures were self-reported making them potentially subject to social desirability bias [108]. Some research, however, suggests self-report measures of PA and more objective measures such as treadmill testing may be in rough concordance [109]. Another concern is the discrepancy between the initial screening of participants, which limited participants to a maximum of 60 minutes of exercise per week, and the amounts of PA reported at T1 (eg, Tx: 53.7 min/wk, SD 73.5; Ctrl: 47.5 min/wk, SD 55.6; Appendix 2), which put many participants near or perhaps over the maximum allowable limits at baseline. Despite all these potential shortcomings, however, Tx participants showed significant improvement compared to the Ctrl group, which is a promising outcome.

Participants were a relatively young population of older adults (ie, M=60 years of age), and they tended to be employed, educated, and frequent computer users with at least a middle-class income. Less educated, lower income, rural, and ethnic populations might be less likely to have Internet in their homes [110], and this approach would obviously be inapplicable for seniors who do not use computers. While seniors are the fastest growing demographic online [43], research is needed to determine if the results of this study generalize to even older generations and to other demographic categories.

The higher attrition rate among treatment participants compared to control participants is another limitation that may have biased the study findings. However, because experimental conditions did not interact significantly with any of the baseline participant characteristics in predicting study attrition, the potential confounding due to differential attrition would appear to be minimal. Furthermore, the use of maximum likelihood estimation for missing data would help to reduce potential biases associated with study attrition.

### Conclusions

Despite limitations, this research demonstrates that a theoretically based stand-alone Internet exercise program that tailors content according to users’ preferences and interests can increase self-reported PA and be well received by sedentary older adults. This type of intervention can be available to users 24/7 on the Internet, making it a potentially cost-effective PA tool that can reach large numbers of people. The results are impressive considering that the study was not conducted as part of a larger health promotion campaign, which might have provided additional support and encouragement for the participants and which might have decreased attrition. Still, more research is needed to understand factors associated with using Internet interventions to maintain engagement in PA over time.

## Multimedia Appendix 1

Demographic information for participants.

## Multimedia Appendix 2

Pretest, posttest, and follow-up descriptive statistics (untransformed values are reported for cardiovascular activities, stretching activities, strengthening activities, and balance).

## Multimedia Appendix 3

CONSORT-Ehealth Checklist V1.6.2 [111].
