# Supplementary material for: Web-based Intervention to Promote Physical Activity by Sedentary Older Adults: Randomized Controlled Trial
Source: J Med Internet Res. 2013 Feb 5;15(2):e19. doi: 10.2196/jmir.2158 (PMC3636271; doi:10.2196/jmir.2158)
Supplement: Supplementary file 1 [file jmir_v15i2e19_app1.pdf]

Appendix 1. Demographic information for participants.

		All cases (n = 368)			Treatment (n = 178)			Control (n = 190)			
		M[a]	SD[b]	%	M	SD	%	M	SD	%	*P* value[c]
**Age (years)**		60.3	4.9		60.3	4.7		60.3	5.1		.948
**Body Mass Index (kg/m²)**		28.9	6.7		29.1	7.3		28.8	6.0		.649
**Gender**											.512
	Male			30.6			28.9			32.1	
	Female			69.4			71.1			67.9	
**Race**											.034
	African American			12.2			16.3			8.4	
	Asian American			11.4			10.7			12.1	
	Caucasian			59.0			53.4			64.2	
	Latino/ Hispanic			9.5			9.0			10.0	
	Native American			1.9			2.2			1.6	
	Pacific Islander			1.1			1.1			1.1	
	Mixed race			3.5			6.7			0.5	
	Other			1.4			0.6			2.1	
**Education**											.808
	Grade school or less			0.5			1.1			0.0	
	Some high school			0.5			0.6			0.5	
	High school graduate			16.5			16.9			16.1	
	Some college			30.2			30.9			29.6	
	College graduate			34.1			34.3			33.9	
	Graduate/ Professional			17.3			15.7			18.8	
	Trade School			0.8			0.6			1.1	
**Employment status**											
	Employed			57.5			61.8			53.5	
	Unemployed			42.5			38.2			46.5	
**Annual family income**											.399
	<$20,000			8.6			9.1			8.2	
	$20,000-39,999			18.6			19.9			17.4	
	$40,000-59,999			28.3			31.3			25.5	
	$60,000-79,999			25.6			23.9			27.2	
	More than			18.9			15.9			21.7	

	$80,000										
**Computer experience**											.510
	Novice			5.8			5.1			6.5	
	Somewhat Experienced			26.7			26.6			26.9	
	Moderately experienced			37.2			37.9			36.6	
	Very experienced			30.3			30.5			30.1	
**Computer use/wk**											.598
	1-2 times			3.0			2.8			3.2	
	3-4 times			7.5			9.1			5.9	
	5-6 times			9.4			9.7			9.2	
	7 times			9.7			9.7			9.7	
	More than 7 times			70.4			68.8			71.9	
**Internet use/wk**											.190
	0 times			0.3			0.6			0.0	
	1-2 times			4.2			4.6			3.8	
	3-4 times			7.5			10.3			4.9	
	5-6 times			8.1			8.6			7.6	
	7 times			8.6			8.6			8.6	
	More than 7 times			71.4			67.4			7.5	
**Email use/wk**											.791
	0 times			1.1			2.3			0.0	
	1-2 times			7.2			6.3			8.1	
	3-4 times			9.7			11.4			8.1	
	5-6 times			8.3			8.0			8.6	
	7 times			7.5			7.4			7.6	
	>7 times			66.1			64.6			67.6	

[a] M = mean.

[b] SD = standard deviation.

[c] Results of statistical comparing differences between Tx and Ctrl groups.
