# Supplementary material for: Web-based Intervention to Promote Physical Activity by Sedentary Older Adults: Randomized Controlled Trial
Source: J Med Internet Res. 2013 Feb 5;15(2):e19. doi: 10.2196/jmir.2158 (PMC3636271; doi:10.2196/jmir.2158)
Supplement: Supplementary file 2 [file jmir_v15i2e19_app2.pdf]

Appendix 2. Pretest, posttest, and follow-up descriptive statistics (untransformed values are reported for cardiovascular activities, stretching activities, strengthening activities, and balance).

Outcome measure/Condition		T1		T2		T3	
		M	SD	M	SD	M	SD
**Cardiovascular activities (min/wk)**							
	Treatment	53.7	73.5	108.6	72.1	122.1	82.9
	Control	47.5	55.6	83.3	71.1	89.8	78.8
**Stretching activities (min/wk)**							
	Treatment	21	39.6	49.1	48.8	50.9	54.5
	Control	20.9	32.1	29.3	31.4	34.3	39.2
**Strengthening activities (min/wk)**							
	Treatment	22.5	52.9	53.7	53.7	51.5	59
	Control	15.6	33.4	29.5	50.6	31.6	42
**Balance (min/wk)**							
	Treatment	8.4	20.8	35.6	44.1	40.4	57.6
	Control	6.2	19.9	17.4	36.4	18.9	39.4
**Number of physical activities (count/wk)**							
	Treatment	6.4	3.5	9.1	2.8	9	3
	Control	6.4	3.2	7.8	3.5	7.8	3.6
**BMI (kg/m^2)**							
	Treatment	29.1	7.2	28.6	7	27.9	6.8
	Control	28.8	6	28.6	6.3	28.3	6.5
**SF-12 physical**							
	Treatment	-0.01	0.76	0.03	0.66	0.04	0.66
	Control	0	0.82	-0.07	0.87	-0.07	0.84
**SF-12 mental**							
	Treatment	0.7	0.8	0.88	0.7	0.86	0.72
	Control	0.8	0.8	0.71	0.8	0.7	0.84
**Attitudes/ Knowledge**							
	Treatment	4	0.6	4.2	0.5	4.2	0.5
	Control	4.1	0.6	4.1	0.5	4.1	0.5

**Self-efficacy**							
	Treatment	3.5	0.9	3.7	0.8	3.8	0.8
	Control	3.6	0.8	3.5	0.9	3.6	0.8
**Behavioral intentions**							
	Treatment	3.7	1	4	0.9	3.9	0.9
	Control	3.7	0.9	3.5	1.1	3.6	1
**Motivation to exercise**							
	Treatment	3.2	0.9	3.6	0.7	3.6	0.9
	Control	3.1	1	3.3	0.9	3.3	0.9
**Ability to exercise**							
	Treatment	3.6	0.4	3.6	0.4	3.7	0.4
	Control	3.6	0.5	3.6	0.5	3.6	0.5
**Barriers to exercise**							
	Treatment	3.5	0.8	3.8	0.8	3.9	0.8
	Control	3.7	0.8	3.7	0.8	3.8	0.8
