# Supplementary material for: Web-based Intervention to Promote Physical Activity by Sedentary Older Adults: Randomized Controlled Trial
Source: J Med Internet Res. 2013 Feb 5;15(2):e19. doi: 10.2196/jmir.2158 (PMC3636271; doi:10.2196/jmir.2158)
Supplement: Supplementary file 3 [file jmir_v15i2e19_app3.pdf]

CONSORT-EHEALTH Checklist V1.6.2 Report

(based on CONSORT-EHEALTH V1.6), available at [http://tinyurl.com/consort-ehealth-v1-6].

2158

Date completed

9/7/2012 14:32:55

by

Blair Irvine

Internet Intervention to Promote Physical Activity
by Sedentary Older Adults: Results from a Randomized Controlled Trial

TITLE

1a-i) Identify the mode of delivery in the title

Internet Intervention to Promote Physical Activity

1a-ii) Non-web-based components or important co-interventions in title

1a-iii) Primary condition or target group in the title

Sedentary Older Adults

ABSTRACT

1b-i) Key features/functionalities/components of the intervention and comparator in the METHODS section of the ABSTRACT

Objectives. This study evaluated the efficacy of a 12-week Internet intervention to help sedentary older adults over 55 years of age adopt and maintain an exercise regimen.

1b-ii) Level of human involvement in the METHODS section of the ABSTRACT

1b-iii) Open vs. closed, web-based (self-assessment) vs. face-to-face assessments in the METHODS section of the ABSTRACT

1b-iv) RESULTS section in abstract must contain use data

1b-v) CONCLUSIONS/DISCUSSION in abstract for negative trials

INTRODUCTION

2a-i) Problem and the type of system/solution

stand-alone 12-week Internet intervention

2a-ii) Scientific background, rationale: What is known about the (type of) system

Shaping a PA intervention to an older adult audience requires a thoughtful approach because seniors may have decade-old habits and attitudes to change, and they may have functional limitations due to age or medical conditions.

METHODS

3a) CONSORT: Description of trial design (such as parallel, factorial) including allocation ratio

We hypothesized that the intervention would be linked to improvement in the above PA domains and to theoretically relevant mediators of behavior change (e.g., attitudes, self efficacy, behavioral intentions), and that user acceptance would be positive.

3b) CONSORT: Important changes to methods after trial commencement (such as eligibility criteria), with reasons

not applicable

3b-i) Bug fixes, Downtimes, Content Changes

not applicable

4a) CONSORT: Eligibility criteria for participants

The on-line screening questionnaire asked respondents a total of 14 questions about current PA levels (i.e., frequency and duration of exercise), desire to exercise more (i.e., yes/no), demographics (i.e., age, gender, race/ethnicity, employment status, computer use), a working email address, and access to a computer Internet connection.

4a-i) Computer / Internet literacy

4a-ii) Open vs. closed, web-based vs. face-to-face assessments:

Participants were recruited via a mixture of online recruitment strategies (e.g., listservs, advertising on seniornet.org), flyers, newsletters, and announcements supported by service agencies, senior centers and worksites. Interested individuals linked to an information-website, which offered a link to an on-line screening questionnaire to determine eligibility

4a-iii) Information giving during recruitment

4b) CONSORT: Settings and locations where the data were collected

on the web

4b-i) Report if outcomes were (self-)assessed through online questionnaires

The study was a randomized controlled trial on the Internet with three assessments: pre-test (T1), post-intervention at 12 weeks after pre-test (T2), and six-month follow-up (

4b-ii) Report how institutional affiliations are displayed

5) CONSORT: Describe the interventions for each group with sufficient details to allow replication, including how and when they were actually administered

5-i) Mention names, credential, affiliations of the developers, sponsors, and owners

5-ii) Describe the history/development process

5-iii) Revisions and updating

5-iv) Quality assurance methods

5-v) Ensure replicability by publishing the source code, and/or providing screenshots/screen-capture video, and/or providing flowcharts of the algorithms used

5-vi) Digital preservation

5-vii) Access
participants accessed the intervention in their context of choice.
5-viii) Mode of delivery, features/functionalities/components of the intervention and comparator, and the theoretical framework
interactive
theory of planned behavior
read the paper
5-ix) Describe use parameters

5-x) Clarify the level of human involvement

5-xi) Report any prompts/reminders used
weekly email prompts to Tx
prompts to do assessments
5-xii) Describe any co-interventions (incl. training/support)
not applicable
6a) CONSORT: Completely defined pre-specified primary and secondary outcome measures, including how and when they were assessed
We hypothesized that the intervention would be linked to improvement in the above PA domains and to theoretically relevant mediators of behavior change (e.g., attitudes, self efficacy, behavioral intentions), and that user acceptance would be positive.
6a-i) Online questionnaires: describe if they were validated for online use and apply CHERRIES items to describe how the questionnaires were designed/deployed

6a-ii) Describe whether and how “use” (including intensity of use/dosage) was defined/measured/monitored

6a-iii) Describe whether, how, and when qualitative feedback from participants was obtained

6b) CONSORT: Any changes to trial outcomes after the trial commenced, with reasons
not applicable
7a) CONSORT: How sample size was determined
7a-i) Describe whether and how expected attrition was taken into account when calculating the sample size

7b) CONSORT: When applicable, explanation of any interim analyses and stopping guidelines
not applicable
8a) CONSORT: Method used to generate the random allocation sequence
randomized groups generated by the database
8b) CONSORT: Type of randomisation; details of any restriction (such as blocking and block size)
Treatment and Control groups
9) CONSORT: Mechanism used to implement the random allocation sequence (such as sequentially numbered containers), describing any steps taken to conceal the sequence until interventions were assigned
randomized groups generated by the database
10) CONSORT: Who generated the random allocation sequence, who enrolled participants, and who assigned participants to interventions
randomized groups generated by the database
11a) CONSORT: Blinding - If done, who was blinded after assignment to interventions (for example, participants, care providers, those assessing outcomes) and how
11a-i) Specify who was blinded, and who wasn’t
no blinding
11a-ii) Discuss e.g., whether participants knew which intervention was the “intervention of interest” and which one was the “comparator”

11b) CONSORT: If relevant, description of the similarity of interventions
not applicable
12a) CONSORT: Statistical methods used to compare groups for primary and secondary outcomes

Baseline Equivalency and Attrition Analysis
The two experimental groups were compared on baseline characteristics and pretest outcome measures. With respect to baseline characteristics, the only significant difference was obtained for race/ethnicity: compared to the Ctrl participants, Tx participants were less likely to be Caucasian (53% vs. 64%; chi-square [1, N = 368] = 4.46, P = .035). Given this significant difference, the main outcome analysis included race/ethnicity as a between-subjects factor. The two conditions did not differ significantly on any of the 13 numeric outcome measures or the Stage of Change groups.
Over the course of the study, a total of 84 (62 Tx; 22 Ctrl) of the 405 randomized participants were unresponsive to repeated prompts and were dropped from the study, and 19 participants (13 Tx; 6 Ctrl) were removed as fraudulent during the 6-month period between T1-T3 assessments. Of the Tx Group participants, only 145 of the 178 who submitted the T1 assessment logged on to initially use the intervention, and six of those participants did not complete Visit 1. A total of 92 (73.6%) of those completing T3 assessments (i.e., 51.7%) from the T1 Tx Group completed all 12 sessions.
Thus, out of the 178 Tx Group participants at T1, 125 (70.2%) eventually remained in the study to T3. A total of 305 participants (125 Tx Group; 180 Ctrl Group) submitted a T2 assessment, and 302 (125 Tx Group; 177 Ctrl Group) submitted a T3 assessment. Overall, T1-T3 attrition was (368-302) /368 = 17.9%.
A significantly higher attrition rate was obtained for the Tx condition compared to the Ctrl condition (30% vs. 7%; chi-square [1, N = 368] = 32.84, P < .001). In addition, significantly higher rates of attrition were obtained for male versus female participants (25% vs.15%; chi-square [1, N = 360] = 4.51, P = .034), race/ethnic minority versus Caucasian participants (26% vs. 12%; chi-square [1, N = 368] = 32.84, P < .001), and those who reported less frequent baseline computer usage (1-2 times per week = 46%, 3-4 times per week 44%, 5-6 times per week = 12%, 7 time per week = 14%, 8 or more times per week = 15%; chi-square [4, N = 361] = 20.83, P < .001). Participants who dropped out of the study after T1 (n = 65) were also compared to those who continued participating (n = 302) on the pretest outcome measures. Compared to the participants who completed either T2 or T3 assessments, those who dropped out had significantly lower means on the attitudes/knowledge scale (3.8 vs. 4.1; t [365] = 3.26, P = .001) [(i.e., attriters had poorer attitudes/knowledge at T1)] and significantly higher mean levels for the barriers to exercise scale (2.7 vs. 2.4; t [365] = 2.86, P = .005). However, no condition-by-attrition interactions were found to be significant for any of the T1 measures (i.e., attriters did not differ across experimental

12a-i) Imputation techniques to deal with attrition / missing values

Missing Data and Imputation
Rates of missing study outcomes ranged from 0-1% at T1, 18-21% at T2, and 19-22% at T3. The full-information maximum likelihood estimators assume data is at least missing at random (MAR). It is not possible to know for sure that data are MAR, because information about the value of the missing data is not available. However, given the abovementioned significant associations between attrition and study outcomes at baseline the MAR assumption appears less tenable. Therefore, the main outcome analyses were conducted with (a) available data (i.e., “complete cases”, n = 294 to 300, dependent outcome), and (b) one fully-imputed data set that includes all 368 study participants. Since the inclusion of additional predictors in the imputation model can reduce bias and make the MAR assumption more plausible (Allison, 2009; He, Zaslavsky, & Landrum, 2009; Rubin, 1996), in addition to the all study outcomes the imputation model also included all study demographic characteristics.
Sequential regression multiple imputation (SRMI; van Buuren, 2007) was used to generate the dataset using the IVEware software V0.2 (Raghunathan, Solenberger, & Van Hoewyk, 2002). SRMI specifies a multivariate model by separate conditional models for each incomplete variable allowing for imputation of variables with different distributional properties. For the current study three models were specified; a normal linear regression model for continuous variables, a logistic regression model for binary variables, and a generalized logit regression model for variables with more than two categories.
Results indicate the available data approach and imputed data approach resulted in a similar pattern of results. Following the intent-to-treat approach results from the imputed model are reported below.
Program Usage
As mentioned, 125 (70%) of the original Tx Group remained in the 12-week study. The mean number of visits to the website for these individuals was 15.2 visits (SD 9.02). The mean total time spent using the program summed

12b) CONSORT: Methods for additional analyses, such as subgroup analyses and adjusted analyses

As mentioned, 125 (70%) of the original Tx Group remained in the 12-week study. The mean number of visits to the website for these individuals was 15.2 visits (SD 9.02). The mean total time spent using the program summed across all visits was 123.4 minutes (SD 185.98), and the mean time spent per visit was 9.66 minutes (SD 10.48). Participants each accessed an average of 2.92 (SD 4.30) program segments designed to help overcome specific perceived barriers to exercise.
Pretest-Posttest Change
A 2 x 2 (condition by race/ethnic minority status) MANCOVA was conducted on the posttest outcome measures in which the pretest outcome measures were included as covariates. The dependent measures included (a) physical activity measures, (b) SF-12 physical and mental composite measures, (c) BMI, and (d) psychosocial measures (Table 2).

RESULTS

13a) CONSORT: For each group, the numbers of participants who were randomly assigned, received intended treatment, and were analysed for the primary outcome

The two experimental groups were compared on baseline characteristics and pretest outcome measures. With respect to baseline characteristics, the only significant difference was obtained for race/ethnicity: compared to the Ctrl participants, Tx participants were less likely to be Caucasian (53% vs. 64%; chi-square [1, N = 368] = 4.46, P = .035). Given this significant difference, the main outcome analysis included race/ethnicity as a between-subjects factor. The two conditions did not differ significantly on any of the 13 numeric outcome measures or the Stage of Change groups.
Over the course of the study, a total of 84 (62 Tx; 22 Ctrl) of the 405 randomized participants were unresponsive to repeated prompts and were dropped from the study, and 19 participants (13 Tx; 6 Ctrl) were removed as fraudulent during the 6-month period between T1-T3 assessments. Of the Tx Group participants, only 145 of the 178 who submitted the T1 assessment logged on to initially use the intervention, and six of those participants did not complete Visit 1. A total of 92 (73.6%) of those completing T3 assessments (i.e., 51.7%) from the T1 Tx Group completed all 12 sessions.
Thus, out of the 178 Tx Group participants at T1, 125 (70.2%) eventually remained in the study to T3. A total of 305 participants (125 Tx Group; 180 Ctrl Group) submitted a T2 assessment, and 302 (125 Tx Group; 177 Ctrl Group) submitted a T3 assessment. Overall, T1-T3 attrition was (368-302) /368 = 17.9%.
A significantly higher attrition rate was obtained for the Tx condition compared to the Ctrl condition (30% vs. 7%; chi-square [1, N = 368] = 32.84, P < .001). In addition, significantly higher rates of attrition were obtained for male versus female participants (25% vs.15%; chi-square [1, N = 360] = 4.51, P = .034), race/ethnic minority versus Caucasian participants (26% vs. 12%; chi-square [1, N = 368] = 32.84, P < .001), and those who reported less frequent baseline computer usage (1-2 times per week = 46%, 3-4 times per week 44%, 5-6 times per week = 12%, 7 time per week = 14%, 8 or more times per week = 15%; chi-square [4, N = 361] = 20.83, P < .001). Participants who dropped out of the study after T1 (n = 65) were also compared to those who continued participating (n = 302) on the pretest outcome measures. Compared to the participants who completed either T2 or T3 assessments, those who dropped out had significantly lower means on the attitudes/knowledge scale (3.8 vs. 4.1; t [365] = 3.26, P = .001) [(i.e., attriters had poorer attitudes/knowledge at T1)] and significantly higher mean levels for the barriers to exercise scale (2.7 vs. 2.4; t [365] = 2.86, P = .005). However, no condition-by-attrition interactions were found to be significant for any of the T1 measures (i.e., attriters did not differ across experimental condition on the T1 measures).
Missing Data and Imputation
Rates of missing study outcomes ranged from 0-1% at T1, 18-21% at T2, and 19-22% at T3. The full-information maximum likelihood estimators assume data is at least missing at random (MAR). It is not possible to know for sure that data are MAR, because information about the value of the missing data is not available. However, given the abovementioned significant associations between attrition and study outcomes at baseline the MAR assumption appears less tenable. Therefore, the main outcome analyses were conducted with (a) available data (i.e., "complete cases", n = 294 to 300, dependent outcome), and (b) one fully-imputed data set that includes all 368 study participants. Since the inclusion of additional predictors in the imputation model can reduce bias and make the MAR assumption more plausible (Allison, 2009; He, Zaslavsky, & Landrum, 2009; Rubin, 1996), in addition to the all study outcomes the imputation model also included all study demographic characteristics.
Sequential regression multiple imputation (SRMI; van Buuren, 2007) was used to generate the dataset using the IVEware software V0.2 (Raghunathan, Solenberger, & Van Hoewyk, 2002). SRMI specifies a multivariate model by separate conditional models for each incomplete variable allowing for imputation of variables with different distributional properties. For the current study three models were specified; a normal linear regression model for continuous variables, a logistic regression model for binary variables, and a generalized logit regression model for variables with more than two categories.
Results indicate the available data approach and imputed data approach resulted in a similar pattern of results. Following the intent-to-treat approach results from the imputed model are reported below.

13b) CONSORT: For each group, losses and exclusions after randomisation, together with reasons

Page views included a total of 3472 on the information website, 2643 on the first screening-questions page, 2589 responses to the first screening item, 449 responses to the last screening item, with 405 individuals ultimately qualifying for the research.

13b-i) Attrition diagram

14a) CONSORT: Dates defining the periods of recruitment and follow-up

not relevant

14a-i) Indicate if critical "secular events" fell into the study period

14b) CONSORT: Why the trial ended or was stopped (early)

it was over

15) CONSORT: A table showing baseline demographic and clinical characteristics for each group

table 1

15-i) Report demographics associated with digital divide issues

in table 1

16a) CONSORT: For each group, number of participants (denominator) included in each analysis and whether the analysis was by original assigned groups

16-i) Report multiple "denominators" and provide definitions

Thus, out of the 178 Tx Group participants at T1, 125 (70.2%) eventually remained in the study to T3. A total of 305 participants (125 Tx Group; 180 Ctrl Group) submitted a T2 assessment, and 302 (125 Tx Group; 177 Ctrl Group) submitted a T3 assessment. Overall, T1-T3 attrition was (368-302) /368 = 17.9%.

16-ii) Primary analysis should be intent-to-treat

17a) CONSORT: For each primary and secondary outcome, results for each group, and the estimated effect size and its precision (such as 95% confidence interval)

An overall multivariate model was tested at posttest, followed by univariate models for each outcome measure. Partial eta-square was used as the estimate of the effect size; values of .01, .06, and .14 represent small, medium, and large effect sizes, respectively [79]. The multivariate model at posttest was significant in which the Tx participants were found to have large gains compared to the Ctrl participants, F (14, 337) = 4.81, P = .001, eta-square = .17 (Table 3). As can been seen in Table 3, the Tx Group differed significantly from the Ctrl participants on 13 of the 14 outcome measures. The only measure not showing significant T1-T2 change was BMI. The outcome measures with medium effect sizes or larger include cardiovascular exercises min/wk (eta-square = .07), stretching exercises min/wk (eta-square= .07), strength exercises min/wk (eta-square = .11), balance exercises min/wk (eta-square = .09), number of activities (eta-square = .07), behavioral intentions to exercise (eta-square = .10), and motivation to exercise (eta-square = .06). Neither the multivariate main effect for race/ethnicity nor the condition-by-race/ethnicity interaction effect was significa

17a-i) Presentation of process outcomes such as metrics of use and intensity of use

17b) CONSORT: For binary outcomes, presentation of both absolute and relative effect sizes is recommended

An overall multivariate model was tested at posttest, followed by univariate models for each outcome measure. Partial eta-square was used as the estimate of the effect size; values of .01, .06, and .14 represent small, medium, and large effect sizes, respectively [79]. The multivariate model at posttest was significant in which the Tx participants were found to have large gains compared to the Ctrl participants, $F(14, 337) = 4.81$, $P = .001$, eta-square = .17 (Table 3). As can been seen in Table 3, the Tx Group differed significantly from the Ctrl participants on 13 of the 14 outcome measures. The only measure not showing significant T1-T2 change was BMI. The outcome measures with medium effect sizes or larger include cardiovascular exercises min/wk (eta-square = .07), stretching exercises min/wk (eta-square= .07), strength exercises min/wk (eta-square = .11), balance exercises min/wk (eta-square = .09), number of activities (eta-square = .07), behavioral intentions to exercise (eta-square = .10), and motivation to exercise (eta-square = .06). Neither the multivariate main effect for race/ethnicity nor the condition-by-race/ethnicity interaction effect was significa

18) CONSORT: Results of any other analyses performed, including subgroup analyses and adjusted analyses, distinguishing pre-specified from exploratory

An overall multivariate model was tested at posttest, followed by univariate models for each outcome measure. Partial eta-square was used as the estimate of the effect size; values of .01, .06, and .14 represent small, medium, and large effect sizes, respectively [79]. The multivariate model at posttest was significant in which the Tx participants were found to have large gains compared to the Ctrl participants, $F(14, 337) = 4.81$, $P = .001$, eta-square = .17 (Table 3). As can been seen in Table 3, the Tx Group differed significantly from the Ctrl participants on 13 of the 14 outcome measures. The only measure not showing significant T1-T2 change was BMI. The outcome measures with medium effect sizes or larger include cardiovascular exercises min/wk (eta-square = .07), stretching exercises min/wk (eta-square= .07), strength exercises min/wk (eta-square = .11), balance exercises min/wk (eta-square = .09), number of activities (eta-square = .07), behavioral intentions to exercise (eta-square = .10), and motivation to exercise (eta-square = .06). Neither the multivariate main effect for race/ethnicity nor the condition-by-race/ethnicity interaction effect was significa

18-i) Subgroup analysis of comparing only users

19) CONSORT: All important harms or unintended effects in each group

not applicable

19-i) Include privacy breaches, technical problems

19-ii) Include qualitative feedback from participants or observations from staff/researchers

DISCUSSION

20) CONSORT: Trial limitations, addressing sources of potential bias, imprecision, multiplicity of analyses

20-i) Typical limitations in ehealth trials

Limitations

The current results must be viewed cautiously because we have no evidence that the participants actually engaged in PA or provided accurate information. Additionally,.....

21) CONSORT: Generalisability (external validity, applicability) of the trial findings

21-i) Generalizability to other populations

21-ii) Discuss if there were elements in the RCT that would be different in a routine application setting

22) CONSORT: Interpretation consistent with results, balancing benefits and harms, and considering other relevant evidence

22-i) Restate study questions and summarize the answers suggested by the data, starting with primary outcomes and process outcomes (use)

This randomized effectiveness trial to evaluate the Active After 55 Web program indicates that the intervention positively impacted the physical activity of sedentary older adults, and it was well received. The hypotheses were that the intervention would be linked to changes in the exercise domains of endurance, stretching, strengthening, and balance, and that it would be linked to theoretically relevant mediators of behavior change.

22-ii) Highlight unanswered new questions, suggest future research

Other information

23) CONSORT: Registration number and name of trial registry

NCT01579240.

24) CONSORT: Where the full trial protocol can be accessed, if available

in the manuscript

25) CONSORT: Sources of funding and other support (such as supply of drugs), role of funders

in Acknowledgements

X26-i) Comment on ethics committee approval

x26-ii) Outline informed consent procedures

X26-iii) Safety and security procedures

X27-i) State the relation of the study team towards the system being evaluated
